# “On-Time Autism Intervention”: A Diagnostic Practice Framework to Accelerate Access

**DOI:** 10.3389/fpsyt.2022.784580

**Published:** 2022-02-17

**Authors:** Ashley M. Penney, Jessica Greenson, Ilene S. Schwartz, Annette Mercer Estes

**Affiliations:** University of Washington, Seattle, WA, United States

**Keywords:** autism, autism (autism spectrum disorders), early intervention (EI) services, early autism diagnosis, early autism intervention

## Abstract

It is well-documented that autism can be reliably diagnosed by age two and that early signs emerge most often between 18 and 24 months. However, despite the increased awareness and focus on early diagnosis, the average age of diagnosis is over 4 years old; even later for Black children and those who are Medicaid-eligible. In this paper, we will propose a framework for accurate and accelerated autism diagnosis for children before age three. The proposed framework emphasizes a collaborative diagnostic process, which relies heavily on Birth to Three provider knowledge and expertise. Considerations for next steps are presented. This approach could increase access to diagnosis of young children soon after first signs of autism emerge.

## Introduction

Autism Spectrum Disorder (ASD) is a neurodevelopmental disability that impacts social communication and repetitive behaviors that can be identified within the first 3 years of life (1, 2). In the United States (US), despite the recent focus on early detection and diagnosis of ASD in young children, a practice gap persists that disproportionately affects young children of color (3–6). Timely diagnosis and quick entry into intensive, comprehensive, and developmentally appropriate intervention services has long been recommended following the emergence of symptoms of ASD (7, 8). It is imperative to address the gap between the emergence of ASD and starting ASD-specific intervention to address the developmental needs of young children and their families (9). Prioritizing referral to Birth-to-Three (B-3) early intervention in this diagnostic process may help close this gap (10).

## Early Detection And Health Inequities

Most children show clear signs of ASD (2) by 24 months, with some individuals manifesting characteristics earlier (11, 12). Some of the earliest detectable signs of ASD include lower rates of social smiles, reduced time spent looking at faces, little or no babbling, reduced eye contact, no pointing or sharing interests, and limited verbalizations (13, 14). Despite the emergence of symptoms by 24 months, diagnosis typically occurs after age 4 in the US (1). The average age of diagnosis is later for low-resource and non-White children. Latinx children are diagnosed later than White children and receive fewer services (6). The mean age of diagnosis of Black children is 64.9 months, with Black parents waiting more than 3 years on average from first concern to diagnosis (3). This gap even persists for Black children with intellectual disability; a co-occurring condition that usually results in earlier identification (1). Medicaid-eligibility, as well as racial, ethnic, and socioeconomic factors are also associated with decreased access to timely and accurate diagnosis (15, 16). Non-white children are less likely than White children to have a personal doctor or nurse and non-White parents are more likely to report their doctor does not spend enough time with them, does not make them feel like a partner in care, and is not sensitive to their family values or customs (17). Other factors that may contribute to these continuing inequities include maternal education level (18), providers dismissing caregiver concerns (19), and variability in the implementation of ASD screenings and referral following a positive screen in pediatric offices, suggesting the possible need for more culturally sensitive ASD screening tools (20). Innovative approaches to provide timely diagnosis are needed to ensure access to services for all children and families.

## B-3 Service Delivery System

The B-3 service delivery system, established by Part C of the Individuals with Disabilities Education Act (IDEA), is a federal program that supports states in providing individualized services for infants and toddlers with disabilities and their families (21). This system is well-situated to facilitate a more timely and equitable approach to early detection and early intervention for young children with ASD in the US (10, 22). For additional information on the B-3 system and IDEA see https://sites.ed.gov/idea/

Increased collaboration between B-3, medical providers, and diagnostic clinicians is an innovation that may improve outcomes for pediatric populations. For example, better integration of B-3 and newborn screening programs, programs that serve the same population but have different funding structures, recruitment approaches, and approaches to intervention, may improve services for children with Fragile X syndrome (23). Fragile X and ASD share many characteristics and Fragile X is often initially misdiagnosed as ASD (24). Similarly, systematic coordination of the B-3 system with medical and diagnostic providers may benefit children with ASD. Such integration, although requiring coordination and effort, holds promise for improving equitable access and outcomes for young children with ASD and their families.

20 years ago, the National Research Council recommended that services for children with ASD should begin as soon as ASD is suspected (7). This goal has not yet been achieved for many children, especially children of color and those with fewer resources (1, 3, 6, 15, 16). Innovations in the diagnostic process are needed so that entry into ASD-specific services as soon as symptoms emerge is no longer considered *early* intervention, but rather is considered *on-time* intervention.

## Shifting to “On-Time” Identification and Diagnosis

The ‘On-Time Autism Intervention' project (OTAI) is a collaboration at the University of Washington (UW) in Seattle, Washington lead by the UW Autism Center and UW Haring Center for Inclusive Education. OTAI staff consists of researchers, clinicians, and educators with expertise in ASD diagnosis and intervention for young children. The goal of OTAI is to develop, with community partners, innovative strategies to increase equitable access to “on-time” diagnostic and ASD-specific intervention services for young children and their families.

The OTAI identification and diagnosis practice framework consists of practice recommendations in five areas: (1) universal screening by B-3 staff, (2) supporting parental orientation toward diagnosis, (3) collaborative referral to diagnostic evaluation, (4) accelerated diagnostic evaluation, and (5) seamless transition to ASD navigation and intervention.

To develop the framework, OTAI staff engaged community B-3 partners by visiting B-3 centers, meeting with and observing the work of B-3 providers. From there, a preliminary collaborative, accelerated diagnostic framework was developed and tested with a partner B-3 organization and revised based on observations and feedback from B-3 providers, parents, and OTAI staff. The framework employs reliable and valid clinical measures in the diagnostic process. The measures considered the ‘gold standard' include the Autism Diagnostic Observation Schedule (ADOS) (25), to directly assess ASD characteristics, the Autism Diagnostic Interview (ADI-R) (26) to provide parent-report of ASD characteristics, a standardized developmental measure to determine developmental level [e.g., Battelle Developmental Inventory (27)] and a measure of adaptive functioning [e.g., the Vineland Adaptive Behavior Scale, 3; VABS-3 (28); or Adaptive Behavior Assessment System-III; ABAS-III (29)] (30–32). However, no specific measure is intended to super cede the clinical judgement by an experienced clinician; thus, clinical judgement is considered the ultimate 'gold standard' for ASD diagnosis. As such, the OTAI diagnostic framework includes a developmental interview with the caregivers that covers the same domains as the ADI-R, an ASD evaluation using the ADOS, and standardized testing of the child. The most important innovation of the framework, is instead of redoing the standardized developmental assessment in the diagnostic evaluation, we utilize the developmental testing conducted by the B-3 team. This functionally accelerates the diagnostic process. But also, by combining information across organizations and practitioners, a collaborative process is initiated that fits within the context of the publicly funded B-3 system.

The OTAI framework is informed by efforts over the past decade to help primary care physicians, B-3 early interventionists, and psychologists learn and implement universal screening for ASD and reduce health inequities (33). A fundamental framework tenet is that when there are developmental concerns about a child, *all roads should lead to B-3 programs as the first stop*. This means that whoever raises concerns about ASD or developmental characteristics associated with ASD; pediatrician, parent, day care provider, or extended family member, the child should be referred to B-3 for evaluation for services. We propose that the following practices, implemented by B-3 centers, can reduce the gap between emerging ASD and accessing ASD services.

## Practice 1: Universal Screening by B-3 Staff

### Conduct ASD Screening

Based on existing recommendations for universal ASD-specific screening for primary care medical providers (8) and leveraging the broad reach of the B-3 system, we recommend that B-3 organizations ensure ASD screening is carried out for all children during their eligibility assessment. Specifically, B-3 providers may either:

- Review existing pediatric ASD screening records prior to B-3 evaluation, OR
- If screening has not yet been conducted, or is not made available, B-3 centers should intentionally screen all children for ASD at their eligibility intake meeting.

**Figure 1:**
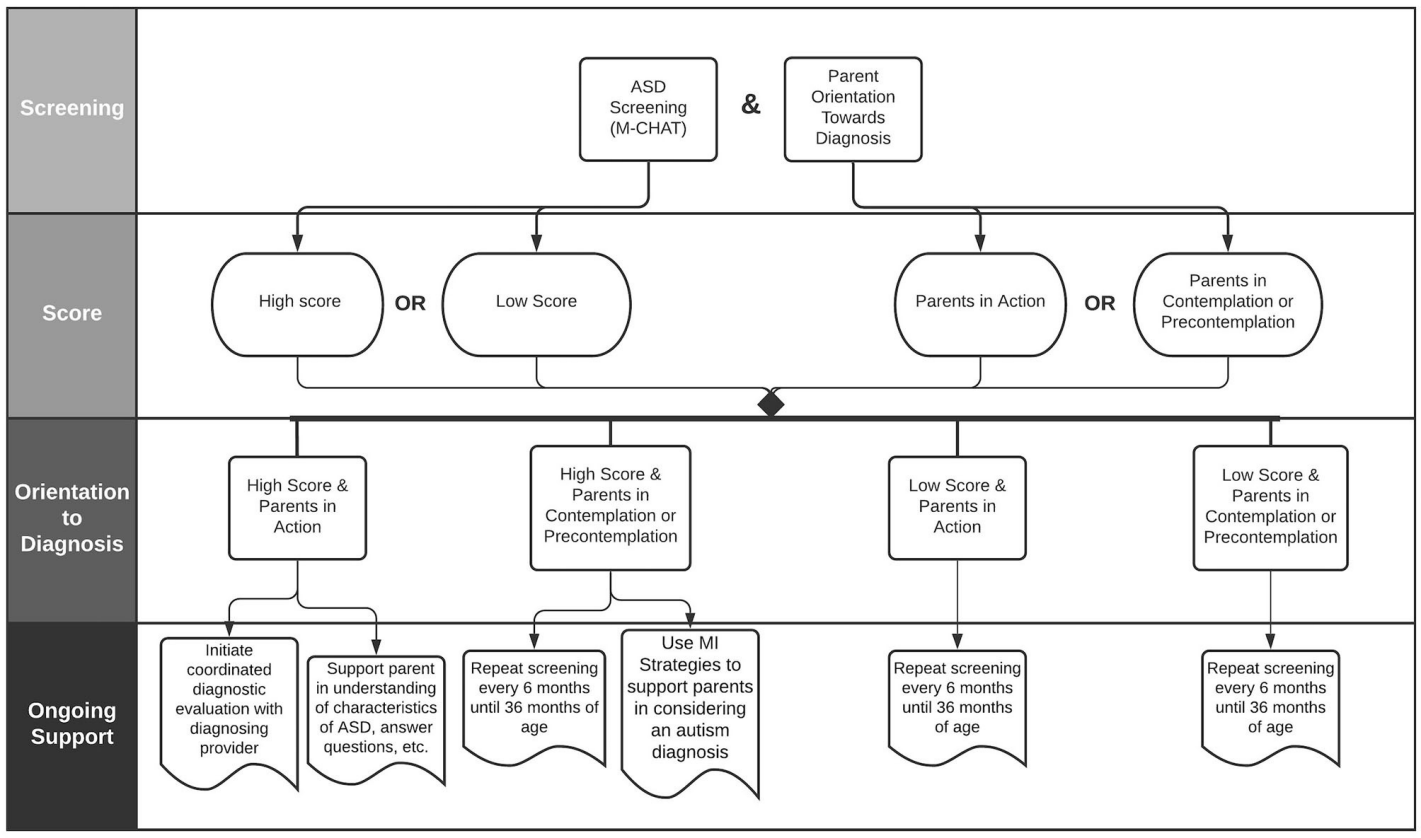
Parent orientation toward diagnosis flowchart.

Standardized developmental testing, including parent reports, is conducted as part of the B-3 eligibility assessment. The OTAI model suggests universal screening, a novel addition to the standard B-3 assessments and questionnaires. B-3 staff should:

- Administer and score the Modified Checklist for Autism in Toddlers (M-CHAT-R/F), using the standardized follow-up questions (34) or
- Review results of any recent M-CHAT screening from a community provider (see Figure 1).
  - If the M-CHAT score indicates further ASD evaluation is warranted, the B-3 provider should assess the parent's orientation toward a diagnosis
  - If the M-CHAT does not indicate further evaluation is warranted, B-3 staff should:
    ∗ Determine whether direct observation or reports from collateral contacts warrant further ASD evaluation.
    ∗ If there is no need for further assessment, B-3 staff should make note of the administration date, continue surveillance for ASD signs, and repeat the M-CHAT every 6 months until 36 months of age (35, 36).
    ∗ If at any point concerns about ASD are raised by parents or B-3 team, the B-3 provider should initiate the M-CHAT screening process as above.

### Introduce the Idea of ASD

The OTAI framework proposes that services should be adapted to meet the needs of parents with different orientations toward receiving, understanding, and acting on diagnostic information. The OTAI framework is designed to help B-3 staff provide support consistent with parental orientation to a diagnosis to move the parent toward engagement with ASD-specific intervention when the time is right for them and in a way that is responsive to parental concern.

Parental responses to early signs of ASD and referral for a diagnostic evaluation range from hesitancy due to fear of labels or stigma, to defensiveness when concerns are brought up by providers, to acceptance or feelings of validation (37, 38). When a child shows characteristics of ASD, the OTAI model suggests the B-3 provider should individualize a plan for working with parents. The first step is to determine the level of caregiver concern about the child and orientation toward diagnosis. The Stages of Change Theory (39) and Motivational Interviewing (MI) strategies (40) have been incorporated into the OTAI framework to help families prepare for diagnostic evaluations when they are ready. These models have been applied in parent coaching for ASD (41) but, to our knowledge, have not yet been applied to support parents in the initial screening and diagnostic referral process.

Parents respond to screening results differently depending upon their orientation toward diagnosis.

- Action: Some parents have been thinking and learning about ASD and are waiting for a provider to suggest they seek out a diagnostic appointment.
- Contemplation: Some parents may be open to the possibility of ASD but are weighing the pros and cons and are still not quite ready to act and schedule a diagnostic evaluation.
- Precontemplation: Some parents may be adjusting to the developmental concerns that led them to B-3 services and have not considered the idea that their child may have ASD. These parents are unlikely to schedule a diagnostic evaluation and if they do, they may not be comfortable with the diagnosis or with seeking ASD-specific services.

## Practice 2: Prepare for Difficult Conversations and Support

If concerns about ASD are indicated on the M-CHAT, but parents are contemplating or not yet contemplating a diagnosis, clinicians should support parental orientation toward a diagnosis and intervention by preparing for difficult conversations and engaging families in conversation.

- Prepare for difficult conversations: B-3 providers should prepare to engage parents using MI strategies to collaboratively explore the ASD characteristics that need further evaluation and to highlight a child's strengths in addition to behavioral and developmental concerns (42).
- Engage in conversations: B-3 providers can engage in ongoing conversations with families about child developmental concerns and positive ASD screens thereby avoiding the pitfall of implicitly or explicitly encouraging families to “*wait and see*.” B-3 providers are experts at supporting families. Sharing additional information about ASD and engaging families in these conversations should aim to support parental orientation toward diagnostic evaluation.
- Parents in the action stage may benefit from:
  - Information about local diagnosing providers
  - Ongoing discussion and information specific to their child
  - Help scheduling and completing the diagnostic evaluation
  - Post-diagnosis navigation support.
- Parents in the contemplation and precontemplation stages may benefit from:
  - Discussing the behaviors on the M-CHAT that suggest ASD
  - Opportunities to ask questions about the assessment, their child's behavior, and other concerns.
  - Conversations about ASD using MI strategies to support orientation towards diagnostic evaluation over the first few months in B-3 services.

In collaboration with B-3 partners, OTAI developed a tool to measure parental orientation towards diagnosis and collect other information relevant to the diagnostic process. This B-3 provider-rated form (available upon request) is designed to support the B-3 provider in tailoring discussions to the parental level of concern and, once a parent decides to pursue a diagnosis, to facilitate efficient and relevant conversations between the B-3 team and the diagnosing clinician. Utilizing the knowledge and expertise of the B-3 provider specific to each family and child is a novel contribution that could support both parents and diagnosing providers when it comes to accelerating access to timely diagnosis. Information for the diagnostic clinician includes:

- Services the child is currently receiving through B-3 agency
  - Child skills and needs in relevant developmental domains (communication, play, social, sensory behaviors, restricted/repetitive behaviors)
- B-3 team impressions of likelihood of ASD
- B-3 team impressions of parental concern regarding ASD (see Table 1)
- Child strengths
- Family support needs

**Table 1:** Questions about parent orientation towards diagnosis from ‘Birth-3 Team Input' Form.

***Instructions:* Please circle all that apply. Use additional space provided to answer each question**.
1. **Parent's Overall Level of Concern about their child and ASD**: Mild/Minimal, Moderate, High
2. **How is family approaching an autism evaluation and possible diagnosis**: Hesitant, anxious/nervous, only doing it because told to/not ready, couple is in conflict-one wants this and other does not, they seem to know s/he has ASD and are ready (other—please explain)
3. **How do you anticipate this parent will respond to an ASD diagnosis**: Denial/Refusal to accept this, Sadness Acceptance/positive thinking, Anger, Self-Blame, Worry, Other
4. **Is there anything else that the team thinks would be important for us to know**: (e.g., trauma, homelessness, language barriers, family culture, financial barriers, marital issues, CPS reports, etc.)

## Practice 3: Referral to Collaborative Diagnostic Evaluation

Once a parent decides to pursue a diagnostic evaluation, an innovative collaborative diagnostic model is recommended. The OTAI model suggests the formation of partnerships between B-3 agencies and local diagnosing clinician(s); psychologists, pediatricians, neurologists, or other professionals, consistent with state law. The collaborative diagnostic model delineates roles for the B-3 provider and diagnostic clinicians and weaves these roles together in a meaningful way to best support the family and accelerate the diagnostic process. B-3 referral for a collaborative diagnosis includes two steps:

- Parental release of information to share B-3 records with diagnostic clinician
- Conversation between B-3 and diagnostic clinician to share relevant information about the child and family

## Practice 4: Accelerated Diagnostic Evaluation

The accelerated diagnostic evaluation utilizes recommended practices (32) for accurate, high-quality diagnosis modified for use in a collaborative partnership with B-3 providers to reduce the amount of time and resources required for diagnosis.

- Diagnostic clinician - Pre-meeting data review:
  - Contact family and schedule first diagnostic visit. Diagnosing clinicians should set a goal to complete the first visit as soon as possible and track how long this takes.
  - Contact B-3 provider to discuss case with child's team.
  - Review records and assessment results provided by B-3 team; medical and developmental history, standardized developmental assessment, M-CHAT screening results, and other relevant records.

Following the pre-meeting data review, the diagnostic clinician will conduct a single, 3-h diagnostic appointment consisting of a parent interview, child-parent play observation, ADOS administration, and diagnostic disclosure. The child, caregivers, and B-3 provider should be in attendance. Involving the B-3 provider in the diagnostic evaluation and disclosure as standard practice is novel and facilitates a coordinated transition between diagnosis and post-diagnosis services. The diagnostic disclosure should include examples of observed child behaviors across the core ASD diagnostic categories, intervention recommendations, and individualized family support information.

Following the diagnostic appointment in accordance with existing clinical practice recommendations, the diagnostic clinician will write a diagnostic report to be shared with the family and B-3 intervention team. The report should include behavioral observations, diagnostic information and scores, intervention recommendations (including a prescription for ABA), and family support resources.

## Practice 5: Seamless Transition To ASD Navigation and Intervention

Once a family has received an ASD diagnosis, they should immediately be offered ASD navigation support as part of their ongoing B-3 services. Navigators can be B-3 service providers of any discipline who have additional training in helping families find ASD-specific services and resources. The family should be assigned an ASD navigator and meet quickly (e.g., within a week) to review the evaluation process, psychologist feedback, and recommendations. The primary role of the navigator is to provide parents with follow-up support, information, and resources after diagnosis. Navigators help parents understand their child's ASD characteristics, seek out resources, pursue and evaluate interventions for the child, and process emotions related to the diagnosis.

## Discussion

The OTAI project suggests that, with adequate funding and support, B-3 intervention service systems can serve a critical and novel role in the effort to increase equitable access to timely diagnosis of ASD through community partnerships with diagnosing clinicians. Novel practices are proposed that are in line with accepted clinical ractice and use valid and reliable measures. Telephone surveys conducted with B-3 providers in the state of California found that although 85% of agencies conduct screening for ASD, only 39% offer diagnostic evaluations prior to age three (43). The OTAI framework, based on partnerships and pilot work in the Pacific Northwest of the US, resulted in the diagnosis of over 120 children by a single provider working part time over 2 years. Parents waited less than 3 months for a diagnosis once they were referred.

Studies examining the parent perspective of diagnosis suggest a need for improvement and innovation. When given opportunities to share about their experiences of the diagnostic process, parents highlighted a need for more information and follow-up after diagnosis (37, 44). Similarly, parents indicated a need for more time during diagnostic feedback meetings, additional follow-up visits, and additional resources about ASD (45). Embedding the diagnostic process into the B-3 experience addresses these concerns since children and families are already working with and receiving support from B-3 providers and programs.

There are several limitations and considerations for future work. Our project staff engaged closely with community providers to increase acceptability and feasibility of the referral and diagnostic process. Despite our efforts to ensure a good fit in our region, this may not be applicable to other communities. Finding diagnostic providers to engage in novel, collaborative diagnosis may be challenging due to structural barriers like low insurance reimbursement or lack of providers with expertise in diagnosis of children under age three. Furthermore, there are questions related to costs of training B-3 providers and logistics, such as who will provide the training, that could impede the implementation of this framework. Finally, the OTAI project was small and focused locally. Scaling up the project to meet the complex and different needs of other regions has not yet been tested. Future efforts are needed to engage community partners to make adaptations to the model that will increase acceptability and feasibility. Additionally, well-designed implementation research is needed to better understand the impact of these practices.

The OTAI Project to date has focused on conducting development field work with partner B-3 agencies. OTAI staff have conducted all diagnoses. The next phase of research will implement the OTAI framework with community diagnostic clinicians and new B-3 agencies. In this next phase, OTAI will provide training but will not provide direct services within B-3 agencies. This will achieve two major purposes, (1) larger scale implementation and testing of the framework, and (2) feedback from community partners to inform novel design and implementation considerations. Further refinement of the OTAI framework will help ensure acceptability and feasibility in a wide range of communities.

For decades large-scale studies have documented delays between first concerns and ASD diagnosis, with greater delays for children of color and those with fewer resources. This research and practice gap suggests the need for innovative advances toward sustainable implementation (46). The OTAI framework presented here is the outcome of an iterative process of co-creating an innovative practice to begin addressing this gap. Children and families received services and the framework was refined collaboratively with providers in the B to 3 system. Within this framework, novel and collaborative concrete actions are proposed which could lead to increased widescale implementation of faster and more equitable diagnosis through the B-3 system, which is available to all regardless of ability to pay. Randomized trials and implementation research methods are needed to further evaluate this model. It is our hope that by reframing ASD diagnosis and intervention to “on-time” rather than “early” and by community replication and refinement of the OTAI practice framework, that other communities may benefit from this work.

## Data Availability Statement

The original contributions presented in the study are included in the article/supplementary material, further inquiries can be directed to the corresponding author/s.

## Author Contributions

AP, JG, IS, and AE devised the project, main conceptual ideas, and framework outline. AP wrote the manuscript with input from all authors. JG implemented and field tested the framework. IS and AE were in charge of overall direction and planning of the project. All authors discussed the results and commented on the manuscript.

## Funding

This project was supported by funding from the Seattle Foundation. Use of facilities and resources supported by: Intellectual and Developmental Disabilities Research Centers (P50HD103524-02).

## Conflict of Interest

The authors declare that the research was conducted in the absence of any commercial or financial relationships that could be construed as a potential conflict of interest.

## Publisher's Note

All claims expressed in this article are solely those of the authors and do not necessarily represent those of their affiliated organizations, or those of the publisher, the editors and the reviewers. Any product that may be evaluated in this article, or claim that may be made by its manufacturer, is not guaranteed or endorsed by the publisher.
